# ULK1 Suppresses Osteoclast Differentiation and Bone Resorption via Inhibiting Syk-JNK through DOK3

**DOI:** 10.1155/2021/2896674

**Published:** 2021-11-15

**Authors:** Yufeng Zhang, Sheng Zhang, Yi Wang, Zhiqiang Yang, Zhe Chen, Nengqiao Wen, Min Yang, Zan Huang, Yuanlong Xie, Lin Cai

**Affiliations:** ^1^Department of Spine Surgery and Musculoskeletal Tumor, Department of Orthopedics, Zhongnan Hospital of Wuhan University, Wuhan 430071, China; ^2^College of Life Sciences, Wuhan University, Wuhan 430072, China

## Abstract

Bone resorption diseases, including osteoporosis, are usually caused by excessive osteoclastogenesis. Unc-51-like autophagy activating kinase 1 (ULK1), a mammalian serine/threonine kinase, may participate in the regulation of bone homeostasis and osteolytic metastasis. In this study, ULK1 expression during osteoclastogenesis was detected with RT-PCR. We knocked down or overexpressed ULK1 through siRNA or lentiviral transduction in bone marrow macrophage (BMM). TRAP and phalloidin staining were performed to detect the osteoclastogenesis activity. Ovariectomized (OVX) mouse model of osteoporosis and a mouse of model osteoclast-induced bone resorption were applied to explore the role of ULK1 in bone resorption in vivo. The results showed that ULK1 expression was downregulated during osteoclast differentiation and was clinically associated with osteoporosis. ULK1 inhibited osteoclast differentiation in vitro. Knockdown of ULK1 expression activated phosphorylation of c-Jun N-terminal kinase (JNK) and spleen tyrosine kinase (Syk). Docking protein 3 (DOK3) was coexpressed with ULK1 during osteoclastogenesis. Downregulation of DOK3 offsets the effect of ULK1 on osteoclastogenesis and induced phosphorylation of JNK and Syk. Activation of ULK1 impeded bone loss in OVX mice with osteoporosis. Additionally, upregulation of ULK1 inhibited osteoclast-induced bone resorption in vivo. Therefore, our study reveals a novel ULK1/DOK3/Syk axis that regulates osteoclast differentiation and bone resorption, and targeting ULK1 is a potential therapeutic strategy for osteoporosis.

## 1. Introduction

Osteoporosis is characterized by bone loss caused by an imbalance of osteogenesis and bone resorption, which can lead to fragility fractures and even death. Bone homeostasis is maintained by balancing osteogenesis and osteolysis, including bone resorption, deposition of matrix proteins, and bone minerals. Bone resorption is a fundamental cellular activity in bone modelling, including bone growth and development. Bone resorption is also associated with bone remodelling and leads to the formation of bone. However, oestrogen deficiency, high levels of glucocorticoids, inflammation, and changes in serum calcium levels all contribute to increase bone resorption [1, 2]. Increasing bone resorption reduces bone mass and disrupts the internal ultrastructure of trabecular bone, thus causing clinical symptoms of osteoporosis. Although many antiabsorbent drugs have been used to inhibit bone loss [3, 4], the efficacy of these drugs remains unsatisfactory partly due to off-target effects and potential side effects, such as osteonecrosis of the mandible, cardiovascular events, and gastrointestinal reactions [3, 5–8]. In recent years, the successful development of bone-forming drugs such as human parathyroid hormone-related polypeptides and antimonoclonal antibodies has opened new avenues for the treatment of osteoporosis. However, their effects last for a very short time, and any discontinuation leads to rapid bone loss and an increased risk of fractures [9, 10]. Clinically, it is important to explore a new therapeutic target for osteoporosis.

Osteoclasts (OC) are the main functional cells responsible for bone resorption. They degrade bone tissue by secreting H^+^, Cl^−^, cathepsin K (CTSK), and matrix metalloproteinases (MMP) in the resorption area [11] and are critical in bone development, growth, repair, and reconstruction. OC are derived from microenvironmental haematopoietic precursor cells, and OC differentiation is determined by macrophage colony stimulating factor (M-CSF) and receptor activator of NF-*κ*B ligand (RANKL). Moreover, mitogen-activated protein kinase (MAPK) regulators, including spleen tyrosine kinase (Syk) and docking protein 3 (DOK3), have been shown to mediate OC differentiation. For example, Syk activation and DOK3 inhibition both increase OC differentiation [12, 13]. Excessive OC activation is common in bone metabolism diseases such as malignant bone tumors, osteoporosis, and autoimmune arthritis. Therefore, OC are promising targets for the prevention of osteoporosis; however, the mechanism of OC differentiation is unclear [14].

Unc-51-like autophagy activating kinase 1 (ULK1) is a mammalian serine/threonine kinase that normally participates in the regulation of bone homeostasis [15] and osteolytic metastasis. Deng et al. reported that ULK1-deficient breast cancer cells promote OC differentiation and function, leading to lytic bone metastasis [16]. Additionally, ULK1 expression in osteocytes decreases with age, which might contribute to the age-related bone loss in senile osteoporosis [15]. Although ULK1 can bind to FIP200, ATG13, and ATG101 to form an autophagy initiation complex, it also exhibits functions unrelated to autophagy. For example, ULK1/2 directly targets glycolytic enzymes to maintain glycolysis during the deprivation of amino acids and growth factors [17]. ULK1 phosphorylates stimulator of interferon genes (STING) to mediate the long-term transcription of innate immune genes [18]. ULK1/2 regulates the output of the endoplasmic reticulum (ER) through SEC16A phosphorylation [19]. ULK1 regulates the ability of cochaperone Cdc37 to coordinate Hsp90-mediated maintenance of the stability and function of protein kinases [20]. Furthermore, the presence of ULK1 regulates the mitogen-activated protein kinase 14 (P38) and c-Jun N-terminal kinase (JNK) pathways [21], which was considered necessary for OC differentiation as an important signal protein in the MAPK. Therefore, further research is needed to uncover the role of ULK1 in osteoclastogenesis and osteoporosis.

In present study, we found that ULK1 expression was downregulated during OC differentiation, and downregulated ULK1 expression was correlated with osteoporosis. Knockdown of ULK1 promotes the OC differentiation, and this process may be due to activating Syk-JNK through inhibiting DOK3. In vivo, ULK1 overexpression or a ULK1 agonist prevented bone loss in mouse osteoporosis models. Our study reveals a novel ULK1/DOK3/Syk axis that regulates OC differentiation, and targeting ULK1 is a potential therapeutic strategy for osteoporosis.

## 2. Materials and Methods

### 2.1. Bioinformatics Analysis

To explore the potential correlation between ULK1 expression and OC differentiation, we analyzed the GSE54779 dataset from the Gene Expression Omnibus (GEO) repository at the National Centre of Biotechnology Information (https://www.ncbi.nlm.nih.gov/geo/) based on the GPL6246 Affymetrix Mouse Gene 1.0 ST Array. The dataset contains three BMM samples treated with RANKL and M-CSF and three BMM samples treated with M-CSF alone. The R and Bioconductor (http://www.bioconductor.org) Affymetrix packages were used to process all raw expression data for standardization. The Linear Array Microarray Analysis (Limma) software package was used to identify the differentially expressed genes (DEGs) in BMM samples treated with M-CSF and RANKL compared to samples stimulated with M-CSF alone. Values of *P* < 0.05 and ∣log2FC | >0.5 were considered thresholds. Hierarchical clustering analyses of mRNAs were performed using R's “pheatmap” software package (version 1.14.0; http://www.bioconductor.org/). To investigate the relationship between ULK1 and DOK3, we analyzed the original gene expression data of GSE56815 from the GEO repository in the National Centre of Biotechnology Information (https://www.ncbi.nlm.nih.gov/geo/) and annotated the genes with the GPL 96 Affymetrix Human Genome U133A Array. This database contains 80 samples from premenopausal and postmenopausal women. GraphPad Prism was used to analyze the correlation between ULK1 and DOK3 gene expression in each sample.

### 2.2. Cell Culture

The mouse macrophage cell line RAW264.7 (ATCC, Cat# TIB-71™) and human kidney epithelial cell line 293T (ATCC, Cat#CRL-3216™) were cultured in DMEM supplemented with 10% foetal bovine serum (FBS) at 37°C in a 5% CO_2_ atmosphere. Primary mouse BMM was isolated from 3- to 4-week-old male Balb/c mice as previously described [22]. Briefly, femurs were dissected under aseptic conditions, the bone marrow cavity was flushed with complete *α*-MEM containing 10% FBS and 1% penicillin-streptomycin, and a single-cell suspension was prepared. Cells were cultured in complete *α*-MEM at 37°C in a 5% CO_2_ atmosphere overnight. M-CSF (R&D Systems, Cat# 416-ML-010) was added to a final concentration of 50 ng/ml, and the cells were cultured for an additional 3 days. For OC differentiation, cells were cultured in complete *α*-MEM containing 50 ng/ml M-CSF and 50 ng/ml RANKL (R&D Systems, Cat# 462-TEC-010) for 6 days. For drug treatment, 10 nM SBI-0206965 (MCE, Cat# HY-16966) or 20 nM LYN-1604 dihydrochloride (MCE, Cat# HY-101923B) was used to inhibit or activate ULK1, respectively.

### 2.3. Knockdown and Overexpression of ULK1 and DOK3

To knock down ULK1 and DOK3, siRNA transfection was performed with a riboFECT CP transfection kit (RiboBio) for 24 h. The target siRNA gene sequences were as follows: ULK1 siRNA: 5′-GAGCAAGAGCACACGGAAA-3′ and DOK3 siRNA: 5′-CAAGATGACATCCAACTGA-3′. The knockdown efficiency was evaluated by quantitative RT-PCR. To overexpress ULK1, we used primers (forward: 5′-ATGGAGCCGGGCCGCCG-3′/reverse: 5′-GTCAGGCATAGACACCACTCA-3′) to amplify the CDS region of ULK1. The CDS of ULK1 cDNA was subcloned into the lentiviral vector pHAGE-puro. Lentiviral packaging and infection were performed as previously described [23]. The plasmids PSPAX2 and PMD2G were used to construct lentiviral vectors by cotransfecting them with overexpression plasmids into 293T cells. After two days, the supernatant was collected for lentiviral transfection. Lentivirus (MOI = 100) was transfected into BMM and RAW264.7 cells with 10 *μ*g/ml polybrene for 24 h. The cells were screened with 2 *μ*g/ml puro for 72 hours. The level of ULK1 overexpression was verified by Western blotting analysis.

### 2.4. TRAP Staining and Phalloidin Staining

TRAP staining was performed to detect OC differentiation ability. Cells were fixed in 4% paraformaldehyde for 20 minutes and soaked in 0.5% Triton-X for 30 minutes. TRAP staining (Servicebio, Cat# G1050) was performed according to the manufacturer's protocols, and cells were fixed in TRAP staining solution for 30 minutes.

For DAPI and phalloidin staining, cells were also fixed in 4% paraformaldehyde for 20 minutes and soaked in 0.5% Triton-X for 30 minutes. DAPI staining (Servicebio, Cat# G1012) and phalloidin staining (Servicebio, Cat# G1041) were performed according to the manufacturer's protocols. Cells were fixed in DAPI and phalloidin staining solution for 10 or 30 minutes.

The results were observed by an inverted fluorescence microscope imaging system (Olympus, IX73). Cells with 2 or more nuclei were counted. The statistics are based on the number of osteoclasts in 8 fields per well.

### 2.5. Quantitative RT-PCR

Total RNA was extracted by using TRIzol reagent (Invitrogen). The RNA was reverse transcribed using Transcriptor Universal cDNA Master Mix (Vazyme, Cat# R111-01), and real-time PCR (Vazyme, Cat# Q711) was performed according to the manufacturer's instructions. The primer sequences were as follows: NFATC1: Forward(F):5′-TATATGAGCCCATCCTTGCCT-3′/Reverse(R):5′-GGCTGCCTTCCGTCTCATAG-3′; RANK: F:5′-CTCCTTGGAAAGCTAGAAGCAC-3′/R:5′-TTCCCTCCCTTCCTGTAGTAAAC-3′; CTSK: F:5′-GCACCCTTAGTCTTCCGCTC-3′/R:5′-GGTCATATAGCCGCCTCCAC-3′; ULK1: F:5′-CCCATCCTAGGCTCTCCTACC-3′/R:5′-AGAGGCCTGAGTCCCAAATG-3′; DOK3: F:5′-GGCTCTGACAAGGGTGTGTTC-3′/R:5′-ACAACCCCACATATGTCTGGG-3′; GAPDH: F:5′-TGAAGGGTGGAGCCAAAAG-3′/R:5′-AGTCTTCTGGGTGGCAGTGAT-3′.

### 2.6. Western Blotting

For Western blotting analysis [24], cell lysates were prepared with an immunoprecipitation lysis solution (Servicebio, Cat# G2038) containing a protease-inhibitor cocktail (MCE, Cat# HY-K0010, 1 : 100) and phosphatase inhibitor cocktail I (MCE, Cat# HY-K0021, 1 : 100). Cell lysates were separated by 10% SDS-PAGE gel, transferred to NC membrane, blocked with 5% milk, and then incubated with primary antibody overnight. On the second day, rinsed the membrane three times with TBST and then incubate with species-specific secondary antibodies for 1 h. For total protein, the phosphorylated protein bands were eluted by the antibody eluaten (Beyotime, Cat#P0025B) and incubated again by total protein antibody as described above. Immunoreactive proteins were detected by a Tanon-5200 (Tanon, 18000856) and analyzed by Image-Pro Plus 6.0. The protein level was normalized to the level of GAPDH, and the phosphorylated protein was normalized to the level of total protein.

The primary antibodies included ULK1 (CST, Cat# 8054, 1 : 1000), DOK3 (Abcam, Cat# ab236609, 1 : 1000), total JNK (CST, Cat# 9252, 1 : 1000), Phospho-JNK (Thr183/Tyr185, CST, Cat# 9255, 1 : 1000), Phospho-Syk (Thy-525/526, CST, 2710, 1 : 1000), Syk (CST, 13198, 1 : 1000), or GAPDH (Proteintech Group In, Cat# 60004-1-Ig, 1 : 4000). GAPDH was used as a control.

### 2.7. Animal Experiments

All animal studies were approved by the Ethics Committee of Wuhan University (protocol# WP2020-08032).

For the osteoporosis mouse model, female BALB/c mice (6 weeks old) were maintained together for 1 week to acclimate to the environment and were randomly divided into three groups: sham, osteoporosis, and osteoporosis treatment with LYN-1604. The mice were anaesthetized with 1-4% isoflurane, and ovariectomy or sham operation was performed. As mentioned before [25], a 0.5 cm single midline dorsal incision was made in the lower back through the skin. Gently free the connective tissue under the skin. Position the ovary under the thin muscle layer, and make a small incision on each side to enter the abdominal cavity of the skin. Expose the fallopian tubes and ovaries. The ovaries were identified and placed back into the abdominal cavity during the sham operation. Ligation was performed around the fallopian tube, and small sterile scissors were used to gently cut off the fallopian tube to remove the ovaries. The rest of the fallopian tube was placed back into the abdominal cavity and sutured layer by layer. For treatment, the mice were administered a ULK1 activator (25 mg/kg, LYN-1604 dihydrochloride, MCE, Cat# HY-101923B) or saline by intraperitoneal injection for two weeks after surgery. All bone analyses were performed at 8 weeks after surgery.

For bone marrow injection experiments, as previously mentioned [26], BMM, which transfected with ULK1 overexpression or control lentivirus, was differentiated for 3 days in the presence of RANKL and M-CSF. Then, they were injected into the femurs (2 × 10^5^ cells, 20 *μ*l) of male Balb/c-nu mice (4 weeks old) every 3 days. Two weeks later, the mice were sacrificed, and the femurs were isolated for further analysis.

### 2.8. Microcomputed Tomography (CT) Analysis

X-ray micro-CT (Skyscan 1276, Bruker Micro-CT) was used to scan the bones. A t protocol was set at an isometric resolution of 7 *μ*m, the aluminium filter was set at 0.25 mm, and X-ray energy settings of 55 kV and 200 *μ*A were used for analysis according to the manufacturer's instructions. For 3D analysis, bone mineral density (BMD) and 3D models were analyzed by the CTAn software (Bruker Micro-CT). The 3D model was adjusted with the CTVol software (Bruker Micro-CT). Trabecular bone parameters in an area from 0.2 mm to 2.3 mm below the growth plate of the femur were measured, including BMD, bone volume to total volume ratio (BV/TV), trabecular bone number (Tb. N), trabecular bone thickness (Tb. Th), and trabecular separation (Tb. Sp).

BV/TV refers to the percentage of cancellous bone volume, which is calculated as the ratio of newly mineralized bone (bone volume) to a given target volume (total volume, TV). Tb. N and Tb. Th represent the number of cancellous bones passing through a unit length and the average thickness of cancellous bone, respectively. These factors are essential for measuring bone growth, and higher values are directly proportional to bone strength. Tb. Sp indicates the average width of the medullary cavity between trabecular bones, and a higher value is inversely proportional to bone strength.

### 2.9. Histological Analysis

For paraffin sections, the femur was fixed in 4% paraformaldehyde for 48 h, decalcified in 10% EDTA for 28 days, and embedded in paraffin. The tissue was sliced (8 *μ*m) with a Leica RM2235 microtome, dewaxed, and subjected to TRAP and H&E staining. The bone histomorphometry was performed as previously described [27, 28].

For frozen sections, the femurs were fixed in 4% paraformaldehyde for 6 h and decalcified as previously described [29]. The samples were further dehydrated with 30% sucrose and embedded in a gelation-based embedding solution after 1 day. We used a Leica CM3050S cryostat to slice the samples (20 *μ*m).

### 2.10. Immunohistochemistry and Immunofluorescence Analysis

For immunohistochemistry, the slices were deparaffinized and treated with citrate buffer solution (pH 6.0) at 95°C 3 times, followed by treatment with 3% H_2_O_2_ at room temperature for 20 minutes. After being blocked with 5% bovine serum albumin (BSA) at room temperature for 1 h, the samples were incubated with primary antibody overnight at 4°C. After the samples were washed, a Polink-2 Plus polymer HRP detection system (ZSGB-BIO, PV6001) was used to incubate the secondary antibody, and DAB (ZSGB-BIO, ZLI-9017) was used for colour development. Haematoxylin was used for nuclear staining. After dehydration and fixation, the slices were scanned by an Aperio VERSA 8.

The primary antibodies included ULK1 (CST, Cat# 8054, 1 : 200) and phospho-JNK (Thr183/Tyr185, CST, Cat# 9255, 1 : 200).

For immunofluorescence analysis, the frozen sections were air-dried and hydrated. After the samples were blocked and permeabilized with 5% BSA and 0.2% Triton-X 100 in PBS for 1 h, the slices were incubated with primary antibodies overnight at 4°C. After being washed, the slices were incubated with a fluorescently labelled secondary antibody for 1 h. The slices were fixed with fluorescent fixation medium containing DAPI (Abcam, Cat# ab104139), and images were captured by a confocal microscope (Leica, SP8) and analyzed by LAS X.

The primary antibodies included ULK1 (CST, Cat# 8054, 1 : 200) and DOK3 (Abcam, Cat# ab236609, 1 : 200).

### 2.11. Statistical Analysis

We used the random number method for random allocation. The investigator was blinded to the group allocation during the experiment. The data are expressed as the means ± SEM. For cell experiments, we used three independent repeated experiments to test the results of the experiment. For animal research, we used five independent experiments for verification. Mice with poor physical condition were excluded before grouping. Statistical significance was analyzed by one-way analysis of variance and Student's *t*-tests. The result was considered statistically significant if the *P* value was less than 0.05.

## 3. Results

### 3.1. ULK1 Is Associated with Osteoclast Differentiation and Bone Loss

**Figure 1:**
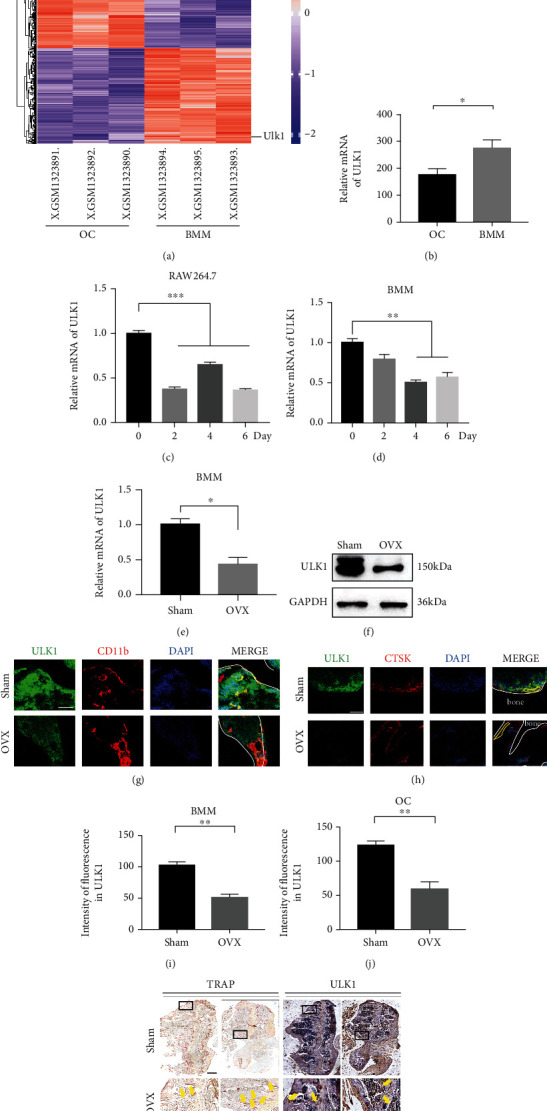
ULK1 is associated with osteoclast differentiation and bone loss. (a) Heat map of differentially expressed mRNA of ULK1 in osteoclasts (OC) and bone marrow macrophage (BMM) from dataset GSE54779 in GEO. (b) Statistical analysis of ULK1 expression between BMM and OC in a heat map. (c) Unc-51-like autophagy activating kinase 1 (ULK1) expression during osteoclast differentiation of RAW264.7 cells. (d) ULK1 expression during osteoclast differentiation of mouse BMM. (e, f) ULK1 mRNA (e) or protein (f) expression in BMM from the sham and OVX groups. (g) Immunofluorescence analysis of ULK1 expression (green) in BMM between the sham and OVX groups. BMM was stained with integrin alpha-M (CD11b) (red). Nucleus was stained with DAPI (blue). The red arrowhead points to BMM. The white line showed the boundary between bone and bone marrow cavity (scale bar, 50 *μ*m). (h) Immunofluorescence analysis of ULK1 expression (green) in OC between sham and OVX mice. OC was stained with cathepsin K (CTSK) (red). Nucleus was stained with DAPI (blue). The yellow line points to OC. The white line showed the boundary between bone and bone marrow cavity (scale bar, 50 *μ*m). (i) Quantification of ULK1 expression in BMM in (g). (j) Quantification of ULK1 expression in OC in (h). (k) TRAP staining (left) and immunohistochemistry (right) of ULK1 in femur sections from the OVX and sham groups. The yellow arrowhead points to OC (scale bar, 250 *μ*m). All data are means ± SEM; ^∗^*P* < 0.05, ^∗∗^*P* < 0.01, ^∗∗∗^*P* < 0.001.

To explore the potential role of ULK1 in OC differentiation, we detected ULK1 expression in cell and mouse models. As shown in Figures 1(a) and 1(b), the mRNA expression of ULK1 decreased in RANKL-induced mouse OC during OC differentiation (GSE54779 from the GEO database). After that, ULK1 downregulation during OC differentiation was confirmed by quantitative RT-PCR analysis of RAW264.7 cells and BMM (Figures 1(c) and 1(d)). ULK1 expression was downregulated at both the mRNA and protein levels in BMM derived from ovariectomized mice compared with BMM derived from control mice (Figures 1(e) and 1(f)). Furthermore, we labelled BMM (red arrow) or OC (yellow line) with integrin alpha-M (CD11b) or CTSK, respectively, and found ULK1 downregulation in BMM from ovariectomized mice with osteoporosis compared to those from sham-operated control mice, and mature OC also had less ULK1 expression (Figures 1(g)–1(j)). At the same time, immunohistochemistry also showed a similar result (Figure 1(k)); the ovariectomized (OVX) group had weaker ULK1 expression than the sham group in the corresponding position of TRAP-positive cells (yellow arrow). These results indicated that ULK1 may be involved in OC differentiation and bone loss.

### 3.2. ULK1 Suppresses Osteoclast Differentiation In Vitro

**Figure 2:**
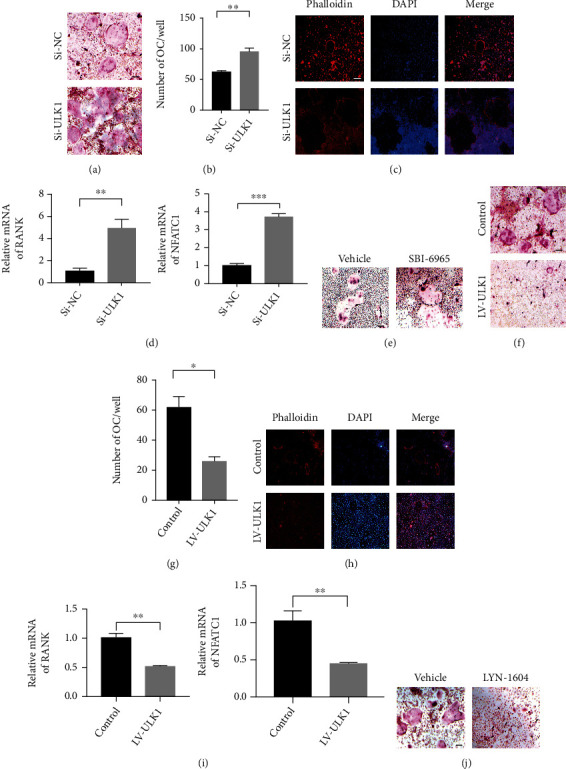
ULK1 suppresses osteoclast differentiation in vitro. (a, b) TRAP staining (a) and quantification (b) to show osteoclast differentiation in control (Si-NC) and ULK1 knockdown (Si-ULK1) cells (scale bar, 100 *μ*m). (c) Actin staining with phalloidin (Cy3, red) in Si-NC and Si-ULK1 OC. Nuclei were stained with DAPI (blue) (scale bar, 100 *μ*m). (d) OC-specific gene expression in Si-NC and Si-ULK1 cells. (e) TRAP staining in vehicle-treated and SBI-6965-treated OC. (f, g) TRAP staining (f) and quantification (g) to show osteoclast differentiation in control and ULK1 overexpressing OC (scale bar, 100 *μ*m). (h) Actin staining with phalloidin (Cy3, red) in control and ULK1 overexpressing OC. Nuclei were stained with DAPI (blue) (scale bar, 100 *μ*m). (i) OC-specific gene expression in control and ULK1-overexpressing cells. (j) TRAP staining in vehicle-treated and LYN-1604-treated OC (scale bar, 100 *μ*m). All data are means ± SEM; ^∗^*P* < 0.05, ^∗∗^*P* < 0.01, ^∗∗∗^*P* < 0.001.

Then, we knocked down ULK1 with siRNA or overexpressed ULK1 through lentiviral transduction in BMM (Figure S1A, B). Neither upregulation nor downregulation of ULK1 expression affected the proliferation of BMM (Figure S1C). As evidenced by TRAP staining and phalloidin staining of actin, si-ULK1-treated BMM exhibited more multinucleated cells than si-NC-treated BMM (Figures 2(a)–2(c)). Consistently, si-ULK1-treated BMM exhibited upregulated expression of OC differentiation genes, including receptor activator of NF-KB (RANK) and nuclear factor of activated T-cells and cytoplasmic 1 (NFATC1), compared to si-NC-treated BMM (Figure 2(d)). Furthermore, the ULK1 inhibitor SBI-6965 increased the formation of TRAP-positive multinucleated cells (Figure 2(e)). In contrast, ULK1 overexpression induced the opposite phenotypes, as evidenced by fewer multinucleated cells, as shown by TRAP staining and phalloidin staining and decreased the expression of OC-specific genes (Figures 2(f)–2(i)). Notably, the ULK1 activator LYN-1604 decreased the formation of TRAP-positive multinucleated cells (Figure 2(j)). These results suggested that ULK1 suppressed OC differentiation in mouse BMM.

### 3.3. Syk-JNK Signalling Mediates the Effects of ULK1 on Osteoclast Differentiation

**Figure 3:**
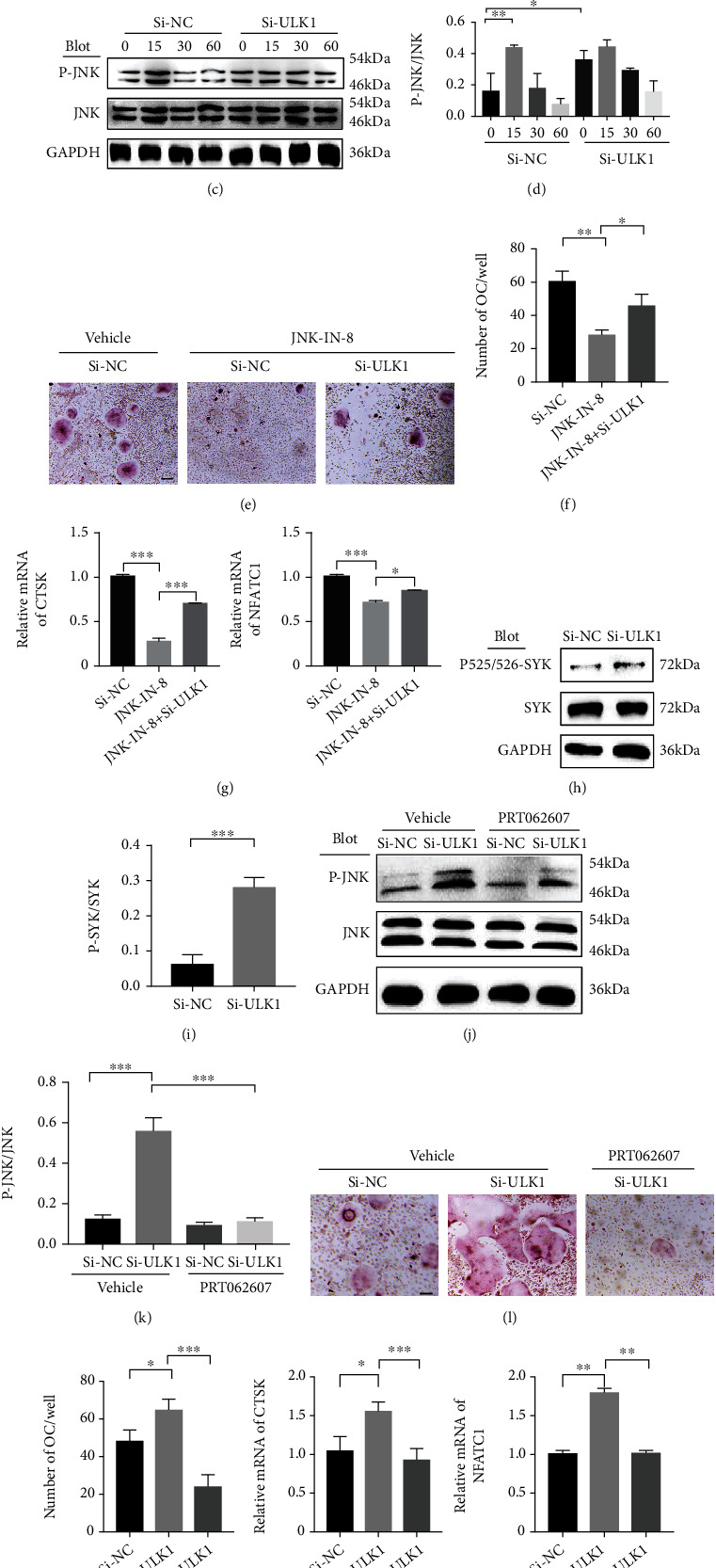
JNK signalling mediates the effect of ULK1. (a, b) Western blotting (a) and quantitative analysis (b) of c-Jun N-terminal kinase (JNK) signalling in RAW264.7 cells overexpressing ULK1 treated with 50 ng/ml of RANKL for 0–60 minutes. (c, d) Western blotting (c) and quantitative analysis (d) of JNK signalling in RAW264.7 cells with ULK1 knockdown treated with 50 ng/ml RANKL for 0–60 minutes. (e, f) TRAP staining (e) and quantification (f) in Si-NC, JNK-IN-8, and Si-ULK1 with JNK-IN-8 OC (scale bar, 100 *μ*m). (g) OC-specific gene expression in Si-NC, JNK-IN-8, and Si-ULK1 with JNK-IN-8 BMM. (h, i) Western blot (h) and quantitative analysis (i) of p-Syk and total spleen tyrosine kinase (Syk) levels in Si-NC and Si-ULK1 RAW264.7 cells. (j, k) Western blot (j) and quantitative analysis (k) of p-JNK and total JNK levels in Si-NC and Si-ULK1 cells treated with or without PRT062607. (l, m) TRAP staining (l) and quantification (m) in BMM of Si-NC, Si-ULK1, and Si-ULK1 treated with PRT062607 (scale bar, 100 *μ*m). (n) The expression of OC-specific genes in BMM of Si-NC, Si-ULK1, and Si-ULK1 treated with PRT062607. All data are means ± SEM; ns *P* > 0.05. ^∗^*P* < 0.05, ^∗∗^*P* < 0.01, ^∗∗∗^*P* < 0.001.

To explore the detailed mechanism by which ULK1 regulates osteoclast differentiation, we further detected the JNK signalling pathway, which has been confirmed to determine OC differentiation in the presence of RANKL [6]. In RAW264.7 cells (considered to be a cell line with a similar function as BMM), overexpression of ULK1 suppressed JNK with 50 ng/ml RANKL (Figures 3(a) and 3(b)). In contrast, knockdown of ULK1 enhanced the activation of JNK (Figures 3(c) and 3(d)). To examine the role of JNK in the effect of ULK1 knockdown on OC differentiation, JNK inhibitor 8 (JNK-IN-8) was used to specifically inhibit JNK signalling. As shown in the figure, JNK-IN-8 treatment suppressed osteoclast differentiation and the expression of osteoclastogenic genes, including CTSK and NFATC1 (Figures 3(e)–3(g)). In addition, we also detected Syk, which is upstream of JNK signalling, and the results showed that si-ULK1 enhanced Syk phosphorylation at Tyr525/526 (Figures 3(h) and 3(i)). PRT062607-mediated Syk inhibition significantly decreased the phosphorylation of JNK and abrogated the si-ULK1 induced promotion of JNK signalling (Figures 3(j) and 3(k)). Furthermore, PRT062607 efficiently reversed the effect of si-ULK1 on OC differentiation and the expression of CTSK and NFATC1 (Figures 3(l)–3(n)). These results suggested that activation of Syk-JNK signalling was involved in the regulation of ULK1 in osteoclast differentiation.

### 3.4. ULK1 Regulates the Activation of Syk-JNK through DOK3

**Figure 4:**
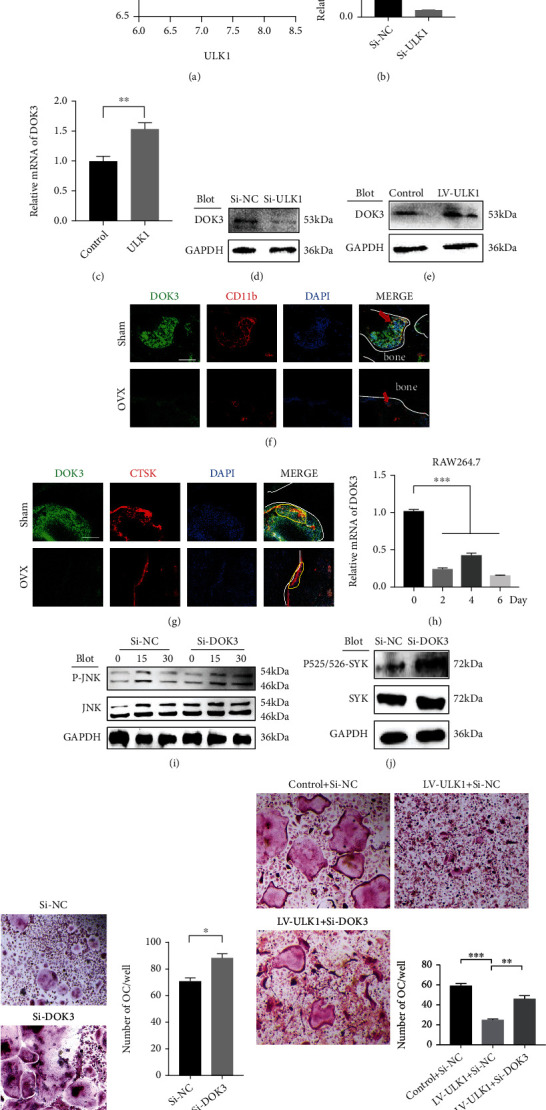
ULK1 regulates the activation of Syk/JNK through DOK3. (a) The correlation of ULK1 and docking protein 3 (DOK3) expression in osteoclast differentiation (GSE56815 dataset). (b) The expression of DOK3 in Si-NC and Si-ULK1 in BMM. (c) DOK3 expression in control and ULK1 overexpressing BMM. (d) Western blotting to detect the expression of DOK3 in Si-NC and Si-ULK1 BMM. (e) Western blotting to detect the expression of DOK3 in control and ULK1 overexpressing BMM. (f) Immunofluorescence analysis of DOK3 expression (green) in BMM between the sham and OVX groups. BMM was stained with CD11b (red). Nucleus was stained with DAPI (blue). The red arrowhead points to BMM. The white line showed the boundary between bone and bone marrow cavity (scale bar, 50 *μ*m). (g) Immunofluorescence analysis of DOK3 expression (green) in OC between the sham and OVX groups. OC was stained with CTSK (red). Nucleus was stained with DAPI (blue). The yellow line marks OC. The white line showed the boundary between bone and bone marrow cavity (scale bar, 50 *μ*m). (h) Expression of DOK3 during osteoclast differentiation of RAW264.7 cells. (i) Western blotting analysis of JNK signalling in Si-NC and Si-DOK3 RAW264.7 cells treated with 50 ng/ml RANKL for 0–30 minutes. (j) p-Syk, total Syk levels of Si-NC and Si-DOK3 in RAW264.7 cells. (k, l) TRAP staining (k) and quantification (l) in Si-NC and Si-DOK3 OC (scale bar, 50 *μ*m). (m) TRAP staining and quantification in control and ULK1 overexpressing and ULK1 overexpressing OC with Si-DOK3 (scale bar, 50 *μ*m). All data are means ± SEM; ^∗∗∗^*P* < 0.001.

DOK3 has been considered a negative regulator of OC differentiation and can inhibit Syk activation [12, 13]. Interestingly, DOK3 and ULK1 were coexpressed during OC differentiation (GSE56815 from the GEO database, *R* = 0.4613, *P* < 0.0001) (Figure 4(a)). We further verified that downregulation of ULK1 expression caused decreased mRNA and protein expression levels of DOK3, and ULK1 overexpression led to the upregulation of DOK3 expression in BMM (Figures 4(b)–4(e)). Moreover, DOK3 expression was downregulated in the BMM (red arrow) or OC (yellow line) of mice with OVX (Figures 4(f) and 4(g)). Meanwhile, compared with BMM, it showed less DOK3 expression in mature osteoclasts which is the same as the change in DOK3 during osteoclast differentiation (Figure 4(h)). We then knocked down DOK3 with siRNA (Figure S1D) and found that DOK3 downregulation also enhanced JNK signalling and Syk phosphorylation, which mimicked the effect of ULK1 downregulation (Figures 4(i) and 4(j)). To test whether DOK3 participates in the effect of ULK1 on Syk activation, we explored the function of DOK3 in OC formation. DOK3 downregulation promoted OC differentiation (Figures 4(k) and 4(l)), and ULK1 overexpression suppressed OC differentiation. Notably, DOK3 knockdown efficiently reversed the inhibitory effect of ULK1 on OC differentiation (Figure 4(m)). Taken together, these findings suggested that DOK3 may mediate ULK1 signalling to Syk, which ultimately affects JNK and OC differentiation.

### 3.5. Activation of ULK1 Alleviates Bone Loss in an OVX Mouse Model of Osteoporosis

**Figure 5:**
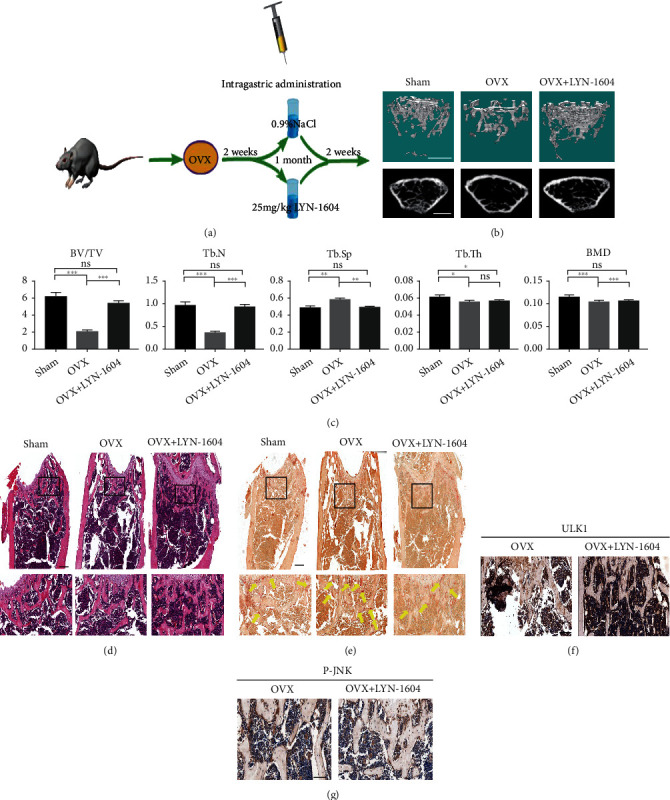
ULK1 activation alleviates bone loss in vivo. (a) Schematic diagram of treatment of OVX mice. (b) Representative *μ*CT images (scale bars, 0.5 mm). (c) Quantification of cortical bone parameters by *μ*CT from sham, therapy, and control mice. Tb. Sp; Tb. N; Tb. Th; BV/TV; BMD (*n* = 5). (d) H&E staining of femur sections from sham, OVX, and OVX mice treated with LYN-1604 therapy (scale bar, 100 *μ*m). (e) TRAP staining of femur sections from sham, OVX, and OVX with LYN-1604 therapy. The yellow arrowhead points to OC (scale bar, 100 *μ*m). (f) Immunohistochemistry of ULK1 in femur sections from OVX mice treated with vehicle and LYN-1604 (scale bar, 100 *μ*m). (g) Immunohistochemistry of p-JNK in femur sections from OVX mice treated with vehicle and LYN-1604 (scale bar, 100 *μ*m). All data are means ± SEM; ^∗^*P* < 0.05, ^∗∗^*P* < 0.01, ^∗∗∗^*P* < 0.001.

To examine whether ULK1 could serve as a potential therapeutic target for bone loss in osteoporosis, an ovariectomized osteoporosis mouse model was established, and then, ULK1 activator (LYN-1604) or saline (Figure 5(a)) was administered by intraperitoneal injection. Micro-CT results showed that oestrogen-deficient mice that were administered ULK1 activator had higher bone mass, bone density, and Tb. N and lower Tb. Sp than OVX mice, while the thickness of the trabecular bone did not change significantly (Figures 5(b) and 5(c)). This result suggested that activating ULK1 can effectively prevent bone loss in oestrogen-deficient osteoporosis. Oestrogen-deficient mice that were administered ULK1 agonists exhibited a more stable trabecular bone structure and more trabecular bone numbers than OVX mice (Figure 5(d)). TRAP staining also showed fewer TRAP-positive cells (yellow arrow) (Figure 5(e)) in the ULK1 activator treatment group. Meanwhile, the increase in ULK1 and the inhibition of JNK signalling in the ULK1 activator treatment group were confirmed by immunohistochemical analysis (Figures 5(f) and 5(g)). Interestingly, no significant difference was found between the ULK1 activation and sham groups. These results suggest that activation of ULK1 may inhibit osteoclast differentiation and alleviate bone loss in an OVX osteoporosis mouse model.

### 3.6. Overexpression of ULK1 Alleviates Osteolysis In Vivo

**Figure 6:**
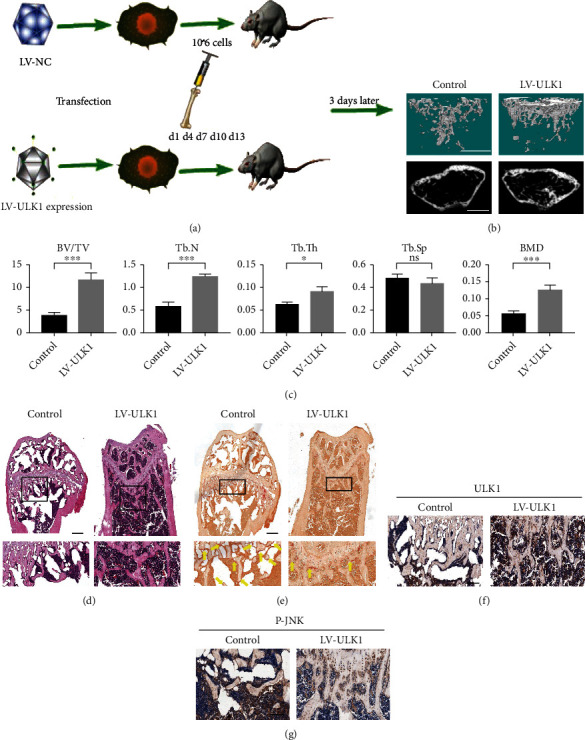
OC overexpressing ULK1 reduces bone resorption in vivo. (a) Schematic diagram of BMM transfer. (b) Representative *μ*CT images (scale bars, 0.5 mm). (c) Quantification of cortical bone parameters by *μ*CT from mice implanted with control and ULK1 overexpressing cells (*n* = 8). Tb. Sp; Tb. N; Tb. Th; BV/TV; BMD. (d) H&E staining of femur sections from mice implanted with control and ULK1 overexpressing cells (scale bar, 100 *μ*m). (e) TRAP staining of femur sections from mice implanted with control and ULK1 overexpressing cells. The yellow arrowhead points to OC (scale bar, 100 *μ*m). (f) Immunohistochemistry of ULK1 in femur sections from mice implanted with control and ULK1 overexpressing cells (scale bar, 100 *μ*m). (g) Immunohistochemistry of p-JNK in femur sections from mice implanted with control and ULK1 overexpressing cells (scale bar, 100 *μ*m). All data are mean ± SEM; ^∗^*P* < 0.05, ^∗∗^*P* < 0.01, ^∗∗∗^*P* < 0.001.

Finally, we established an osteolysis mouse model to explore the role of ULK1 in osteolysis in vivo (Figure 6(a)). Micro-CT detection showed that mice injected with ULK1-overexpressing cells had higher femur bone mass (BV/TV), BMD, Tb. Th, and Tb. N than control mice, whereas the Tb. Sp of the two groups was not significantly different (Figures 6(b) and 6(c)). This effect may be due to the incomplete development of trabecular bone in the growing mice, and there are still large gaps in the medullary cavity. The administration of BMM continuously maintains the development of trabecular bone at a low level, masking the difference in the Tb. Sp. Mice that were administered ULK1-overexpressing cells had higher bone mass than mice that were administered control cells (Figure 6(d), Figure S2A). Compared with the control group, the ULK1 overexpression group had less TRAP-positive cells (Figure 6(e), Figure S2B). Immunohistochemical analysis proved that ULK1 was overexpressed (Figure 6(f)), and that JNK signalling was inhibited (Figure 6(g)). Thus, overexpression of ULK1 may alleviate osteolysis in vivo, which is similar to the results of OVX mice treated with ULK1 activator.

Taken together, these results demonstrate that ULK1 activation confers resistance to bone destruction and oestrogen deficiency-induced osteoporosis. Moreover, it upregulated DOK3, thereby inhibited osteoclastogenesis via Syk/JNK signalling.

## 4. Discussion

Bone resorption is a dynamic process, and OC resorption is balanced with osteoblast formation [30]. The imbalance of these two types of cells can disrupt the integrity of bone structure and cause a series of osteolytic diseases such as osteoarthritis, osteoporosis, and loosening of sterility around implants [31, 32]. The renewal of bones is a process of dynamic balance. Osteoclasts remove the existing bones, and osteoblasts derived from mesenchymal stem cells rebuild bones so that bone remodelling is well orchestrated [33–35]. The bone mass becomes abnormal when the balance is broken. Abnormal activation of osteoclasts leads to multiple bone diseases, including osteoporosis, in postmenopausal women due to oestrogen deficiency and metastatic bone diseases [36, 37]. Although numerous studies have shown that abnormal bone resorption diseases are caused by the abnormal activation of OC, the process of abnormal activation of OC is still controversial [30]. This study revealed the molecular mechanism by which ULK1 regulates OC differentiation and determined that ULK1 is an effective target for the treatment of abnormal bone resorption diseases.

A series of osteolytic diseases are thought to be caused by excessive OC [31, 32]. As mentioned previously, excessive bone resorption is mainly inhibited clinically by regulating OC functions. However, most drugs have off-target effects and potential side effects [9, 10], and some drugs even have withdrawal reactions and serious side effects when they are used for a long time [3, 5–8]. Even for some refractory abnormal bone resorption diseases [38, 39], including some hereditary bone sclerosis or malignant osteoporosis, bone marrow transplantation is required, which brings great pain to the patient. Here, our research showed that upregulation of ULK1 expression rescues the occurrence of excessive bone resorption. LYN-1604 dihydrochloride, used to treat triple-negative breast cancer, is an effective ULK1 activator (EC50 = 18.94 nM) [40]. Our experiments discovered its new function in inhibiting excessive bone resorption. The bone mass, bone density, and trabecular bone number of ovariectomized mice given LYN-1604 were higher than those of control mice and similar to those of sham group. In addition, pathological analysis revealed fewer OC in ovariectomized mice given LYN-1604 than in ovariectomized mice. This demonstrates the feasibility of the use of ULK1 as a therapeutic target for excessive bone resorption diseases.

OC is derived from microenvironmental haematopoietic precursor cells, and their formation and function are determined by M-CSF and RANKL [41]. At the same time, some researchers believe that autophagy also plays an important role in OC differentiation [42, 43]. ULK1 is considered to be a regulator of autophagy [44] and JNK signalling. Considering their role in OC differentiation, we first verified the changes in ULK1 in osteoclast differentiation and disease models. ULK1 expression is downregulated in OC differentiation and abnormal bone resorption diseases. This result is unexpected, which contradicts our previous inferences. Although Arai et al. [45] also observed this change, it did not give a reasonable explanation. This prompted us to further research how ULK1 regulates the process of osteoclast differentiation. Although limited studies have pointed out that ULK1 may be involved in high glucose-regulated osteoclastogenesis [42], these findings were derived entirely from the RAW264.7 cell line as a model of osteoclastogenesis. The study of primary cells is considered necessary and interesting. Therefore, we regulated ULK1 in BMM and found that the downregulation or upregulation of ULK1 expression promotes or inhibits OC differentiation and the expression of related OC genes. We found that JNK signalling changed significantly. In addition, ULK1 regulates JNK signalling pathway in primary astrocytes in traumatic brain injury [21]. That proves our conclusion from another side.

We also found that the Syk/DOK3 signalling axis plays an important role in signal transduction from ULK1 to JNK. DOK3 proteins are adapter or scaffold proteins that are enzyme-inactive. It provides a docking platform for assembling multimolecular signalling complexes and is a negative regulator of JNK signalling. Some studies have pointed out that OC differentiation increases when DOK3 is inhibited or Syk is activated [12, 13]. Moreover, DOK3 can regulate OC differentiation by affecting the Syk and JNK pathways [13, 46], and phosphorylated Syk can activate JNK in macrophages [47]. In our study, we showed that ULK1 inhibited Syk and JNK by regulating the expression of DOK3. Analysis of the GEO database revealed a positive correlation between ULK1 and DOK3, which was later validated by PCR and Western blotting analysis. We then observed that DOK3 expression was reduced in both OC differentiation and OVX samples. These results indicated that DOK3 plays an important role in osteoporosis caused by increased OC differentiation and may be regulated by ULK1. When DOK3 expression was downregulated, JNK and Syk phosphorylation levels were found to show consistent changes with ULK1 regulation. Therefore, we believe that DOK3 may play an important role in the event that ULK1 inhibits Syk activation. At the same time, downregulating the expression of DOK3 rescued the inhibitory effect of upregulated ULK1 expression on OC differentiation. This finding suggests a new regulatory axis in OC differentiation. Nevertheless, some problems still need to be solved. Although DOK3 has been shown to play an important role in the regulation of OC differentiation by ULK1, the mechanism by which ULK1 induces DOK3 expression is still unclear. Some studies have shown ULK1 regulates STING degradation by phosphorylating Ser366 site [18], and STING can regulate transcription factors including IRF3, thereby mediating the transcription of many innate immune genes in macrophages [48–50]. ULK1 may induce DOK3 expression by this way. However, the specific regulatory mechanism still needs to be verified in many studies.

## 5. Conclusion

**Figure 7:**
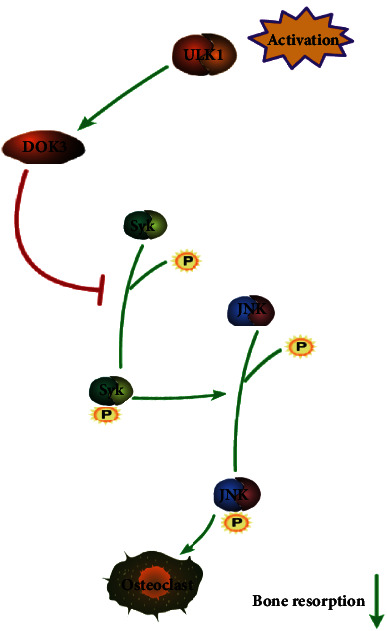
Schematic diagram of the inhibitory effect of ULK1 on osteoclast differentiation and bone resorption activity.

According to our research, ULK1 may regulate OC differentiation via DOK3/Syk/JNK signalling in vitro (Figure 7). Bone mass, bone density, and trabecular bone are important determinants of bone strength in many species, including humans. Changes in the trabecular bone, such as bone density and osteoporosis, affect bone strength and can cause fractures. Additionally, the ULK1 activator LYN-1604 dihydrochloride remedied bone loss in OVX. Thus, activation of ULK1 may be a promising method for treating osteoporosis.

## Data Availability

The data that support the findings of this study are available from the corresponding author upon reasonable request.

## Supplementary Materials

Supplementary Materials Figure S1: identification of regulating the expression of ULK1 and DOK3. (A) Efficiency of Si-ULK1 knockdown. (B) Efficiency of ULK1 overexpression plasmid. (C) MTT in Si-NC, Si-ULK1, lentivirus-NC, and lentivirus-ULK1 overexpression BMMs. (D) Efficiency of Si-DOK3 knockdown. All data are mean ± SEM; ns *P* > 0.05, ^∗∗∗^*P* < 0.001. Figure S2: the statistics of bone histomorphometry. (A) The statistics of BV/TV in Figure 6(d). (B) The statistics of OC. N in Figure 6(e). All data are means ± SEM; ns *P* > 0.05, ^∗^*P* < 0.05. Figure S3: the wells of TRAP staining. (A) The wells of TRAP staining in Figure 2. (B) The wells of TRAP staining in Figure 3. (C) The wells of TRAP staining in Figure 4.
