# Supplementary material for: ULK1 Suppresses Osteoclast Differentiation and Bone Resorption via Inhibiting Syk-JNK through DOK3
Source: Oxid Med Cell Longev. 2021 Nov 15;2021:2896674. doi: 10.1155/2021/2896674 (PMC8608530; doi:10.1155/2021/2896674)
Supplement: Supplementary Materials — Figure S1: identification of regulating the expression of ULK1 and DOK3. (A) Efficiency of Si-ULK1 knockdown. (B) Efficiency of ULK1 overexpression plasmid. (C) MTT in Si-NC, Si-ULK1, lentivirus-NC, and lentivirus-ULK1 overexpression BMMs. (D) Efficiency of Si-DOK3 knockdown. All data are mean ± SEM; ns P > 0.05, ∗∗∗P < 0.001. Figure S2: the statistics of bone histomorphometry. (A) The statistics of BV/TV in Figure 6(d). (B) The statistics of OC. N in Figure 6(e). All data are means ± SEM; ns P > 0.05, ∗P < 0.05. Figure S3: the wells of TRAP staining. (A) The wells of TRAP staining in Figure 2. (B) The wells of TRAP staining in Figure 3. (C) The wells of TRAP staining in Figure 4. [file 2896674.f1.docx]

**Supplementary materials**


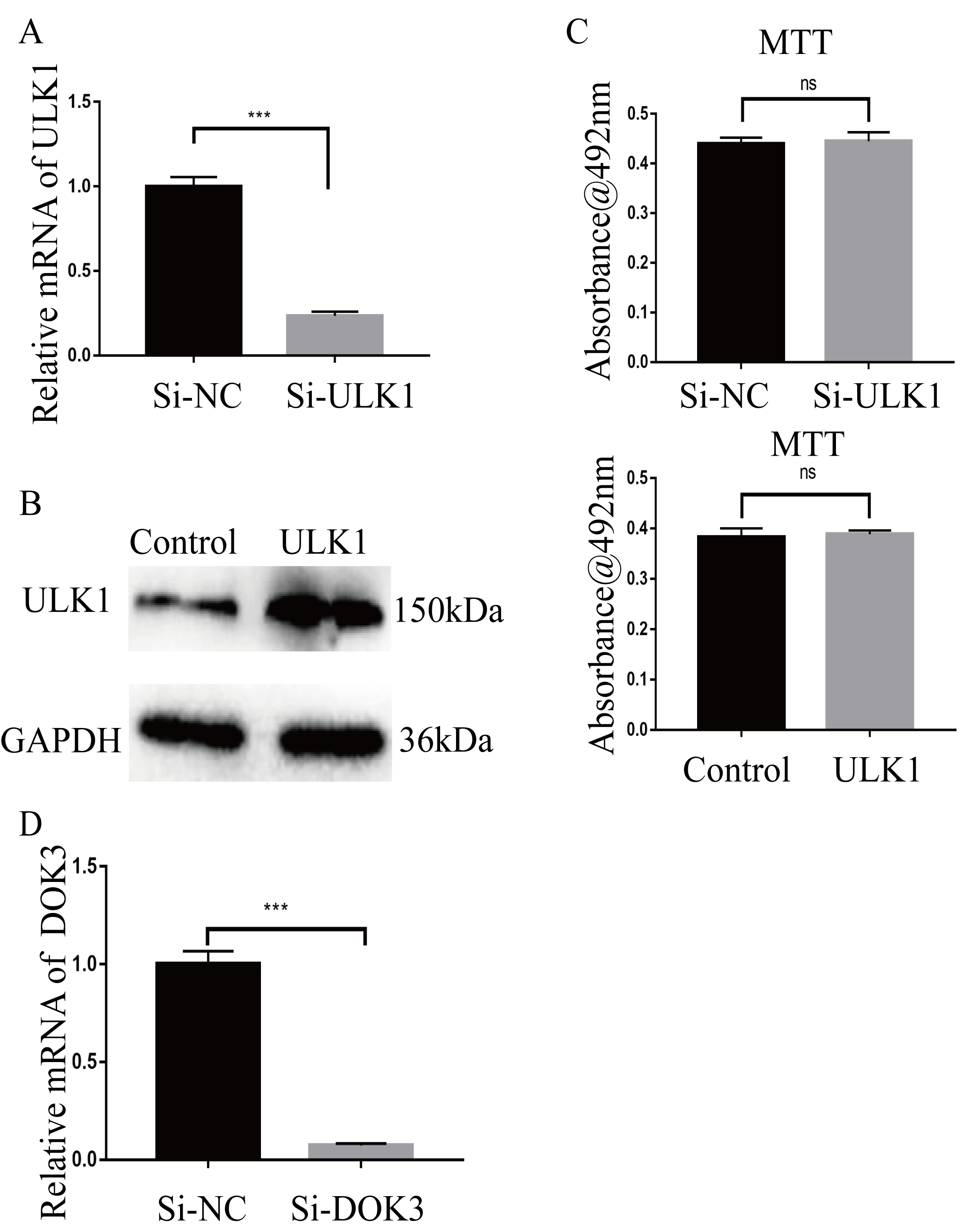


**Figure S1 Identification of regulating the expression of ULK1 and DOK3 (A)** Efficiency of Si-ULK1 knock down. **(B)** Efficiency of ULK1 overexpression plasmid. **(C)** MTT in Si-NC, Si-ULK1, lentivirus-NC and lentivirus-ULK1 overexpression BMMs. **(D)** Efficiency of Si-DOK3 knock down. All data are mean ± SEM; ns P>0.05, *** P<0.001.


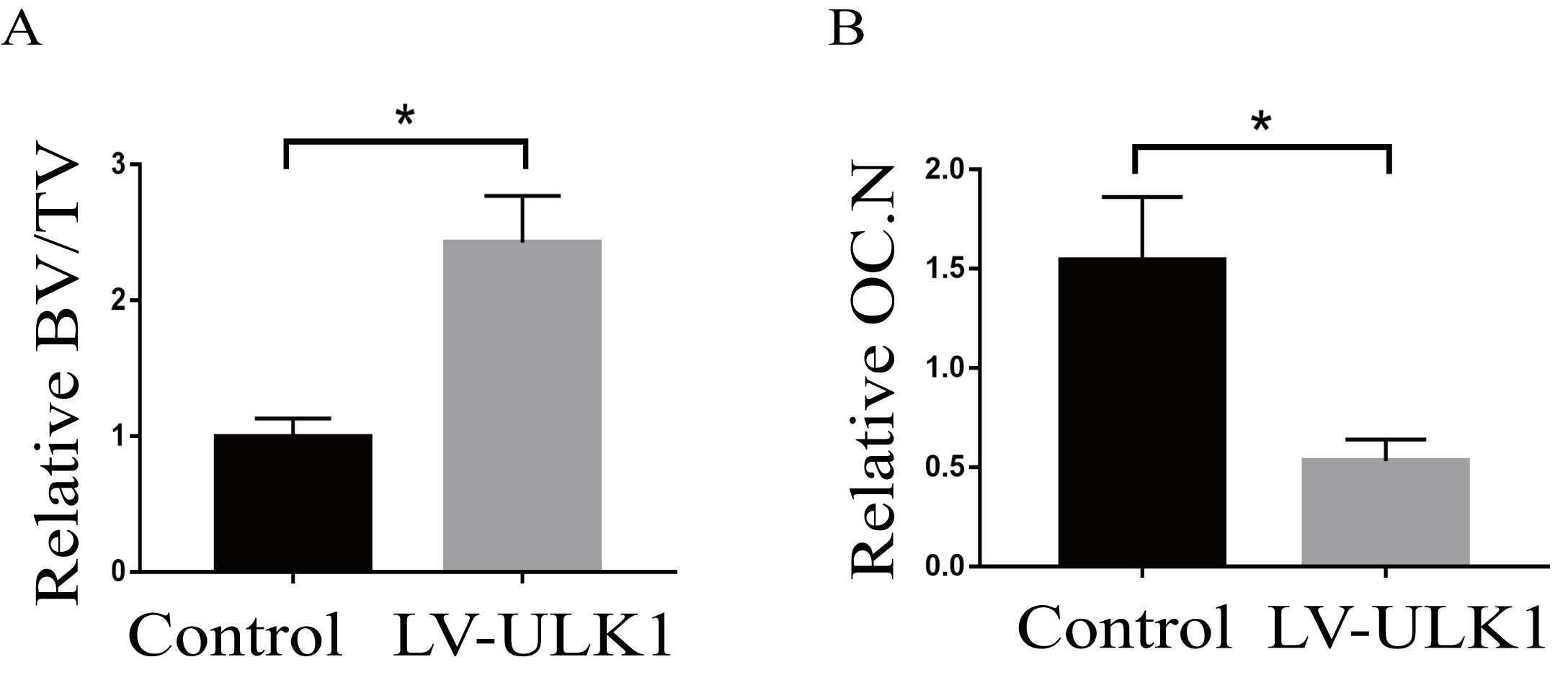


**Figure S2 The statistics of bone histomorphometry (A)** The statistics of BV/TV in Figure 6D. **(B)** The statistics of OC. N in Figure 6E. All data are means ± SEM; ns P>0.05, * P<0.05.


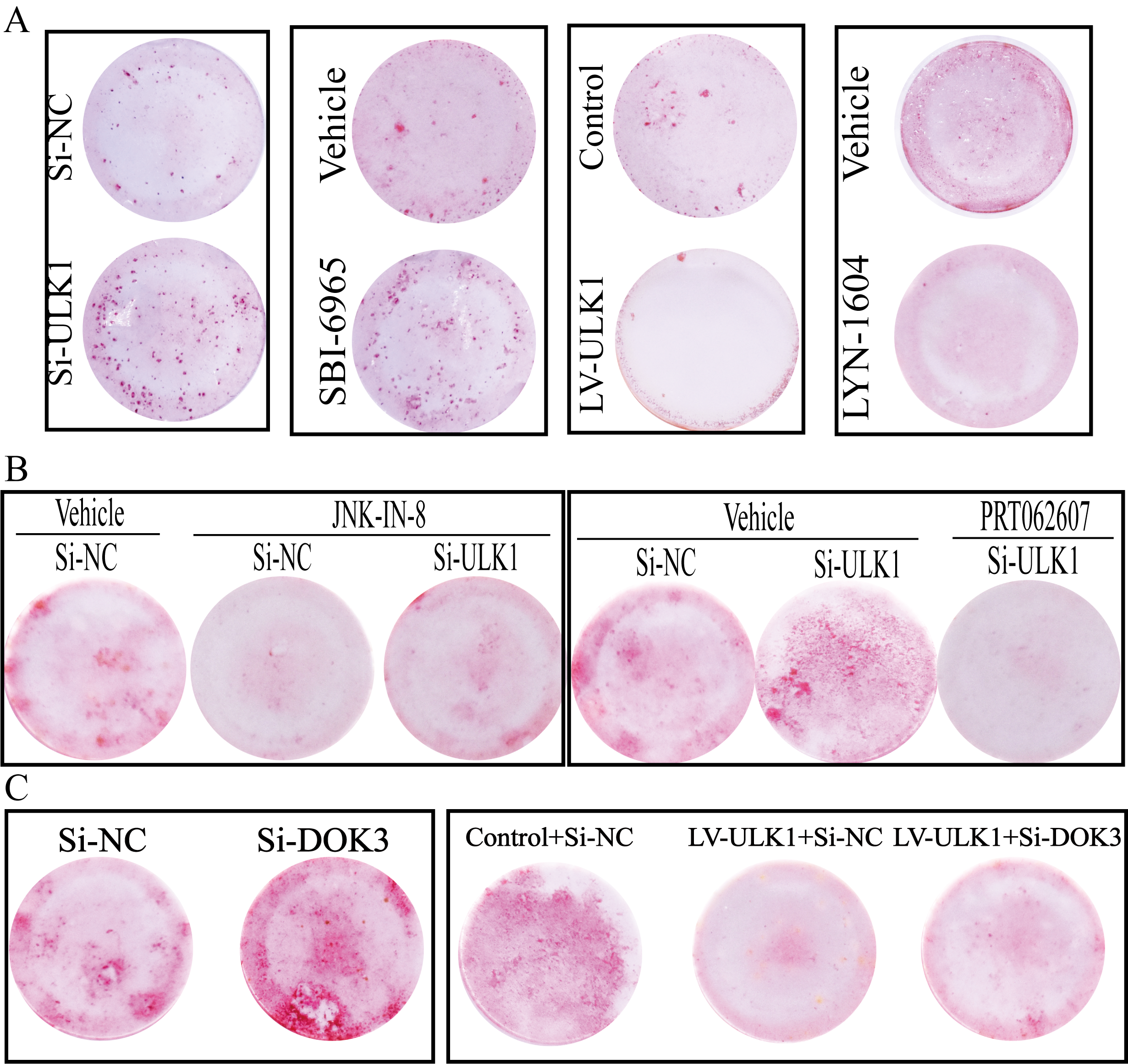


**Figure S3 The wells of TRAP staining (A)** The wells of TRAP staining in Figure 2. **(B)** The wells of TRAP staining in Figure 3. **(C)** The wells of TRAP staining in Figure 4.
